# Unraveling the molecular architecture of a G protein-coupled receptor/*β*-arrestin/Erk module complex

**DOI:** 10.1038/srep10760

**Published:** 2015-06-01

**Authors:** Thomas Bourquard, Flavie Landomiel, Eric Reiter, Pascale Crépieux, David W. Ritchie, Jérôme Azé, Anne Poupon

**Affiliations:** 1 BIOS group, INRA, UMR85, Unité Physiologie de la Reproduction et des Comportements, F-37380 Nouzilly, France; CNRS, UMR7247, F-37380 Nouzilly, France; Université François Rabelais, 37041 Tours, France; IFCE, Nouzilly, F-37380 France; 2 INRIA Nancy, 615 Rue du Jardin Botanique, Villers-lès-Nancy, 54600 France; 3 Bioinformatics group - AMIB INRIA - Laboratoire de Recherche en Informatique, Université Paris-Sud, Orsay, 91405 France

## Abstract

*β*-arrestins serve as signaling scaffolds downstream of G protein-coupled receptors, and thus play a crucial role in a plethora of cellular processes. Although it is largely accepted that the ability of *β*-arrestins to interact simultaneously with many protein partners is key in G protein-independent signaling of GPCRs, only the precise knowledge of these multimeric arrangements will allow a full understanding of the dynamics of these interactions and their functional consequences. However, current experimental procedures for the determination of the three-dimensional structures of protein-protein complexes are not well adapted to analyze these short-lived, multi-component assemblies. We propose a model of the receptor/*β*-arrestin/Erk1 signaling module, which is consistent with most of the available experimental data. Moreover, for the *β*-arrestin/Raf1 and the *β*-arrestin/ERK interactions, we have used the model to design interfering peptides and shown that they compete with both partners, hereby demonstrating the validity of the predicted interaction regions.

Most G Protein-Coupled Receptors (GPCR) have been demonstrated to transduce extracellular signals through both G protein-dependent and G protein-independent pathways. Amongst the G protein-independent pathways, those depending on the *β*-arrestin scaffold proteins have been found for multiple GPCRs^1^,^2^. *β*-arrestins 1 and 2 interact with the activated receptor, and this phenomenon is concomitant with the ending of G protein-dependent signaling. *β*-arrestins also interact with clathrin, resulting in the internalization of the whole complex. Depending on the phosphorylation state of the receptor, this complex is further directed to either desensitization or signaling^3^. Large-scale studies have shown that both *β*-arrestins interact with many different protein partners, and that these interactions depend on the *β*-arrestin activation state^4^. Many of the known partners play crucial roles in GPCR signaling, such as the mitogen-activated protein kinases (MAPKs) ERK, p38 and JNK as well as c-Src, PTEN, Akt, PI3K and many others^5^. Since no enzymatic activity has ever been detected for *β*-arrestins, their role is probably to assemble signaling modules, allowing the different elements to remain in close contact for long periods, thus modifying the dynamics of biochemical reaction cascades.

This is notably the case for the Raf/Mek/Erk cascade. Indeed, this cascade exists in the cytoplasm, and is triggered by second messengers following the activation of Gα subunit. In addition, these three proteins have been shown to interact directly with *β*-arrestins^6^,^7^,^8^. Initially demonstrated with the *β*2-adrenergic receptor, G -protein-independent ERK phosphorylation is now generalized to many other receptors^2^,^3^.

Since many different proteins compete to interact with *β*-arrestins, it is essential to determine which partners can interact simultaneously and which are mutually exclusive. To achieve this goal, one way is to test experimentally different combinations of purified partners. This process is time-consuming and the available experimental methods have many drawbacks. Moreover, the combinatorial complexity which would have to be explored precludes a systematic study. A second way is thus to predict the geometry of the possible assemblies using protein-protein docking and then to validate experimentally the predictions. More generally, such a modeling pipeline would allow to understand the functioning of so-called “hub” proteins. Apart from *β*-arrestins, other essential proteins like PP2A (protein phosphatase 2A), 14-3-3, p53 or *β*-catenin are also considered as hubs.

The principle of protein-protein docking is to determine the best association geometry from the 3D structures of the individual partners. In contrast to protein-ligand docking methods, because of the very large number of atoms in the ligand, protein-protein docking methods do not have a sub-ångström precision, and solutions having an RMSD (Root Mean Square Deviation) under 10 Å with the native conformation are generally considered satisfactory, and will be denominated near-native. A second aspect that should be highlighted is that protein-protein docking methods try to predict the best assembly of two proteins, but do not predict whether this assembly exists or not, and scores are generally not correlated with affinity. To this aim, a very large number of putative conformations of the assembly is generated (typically a few billions) and scored, to extract a reduced number of highly probable conformations (from 5 to 20). The performance of a docking algorithm is determined by its ability to place near-native conformations within this final reduced set of conformations. Because we are interested in predicting the assembly of transient multi-partner complexes, we have developed PRIOR (Protein-Protein cOmplexes 3D structure pRediction), which allows to generate such models in a reduced time (each docking experiment requires a few hours of computation time), with high accuracy (a near-native solution is found in most cases), although giving coarse-grain models. The guideline is to allow the rapid prediction of large macromolecular assemblies that can be further validated, either by extracting data from literature, or experimentally.

We have used this new method to model the 3D arrangement of the *β*2-adrenergic receptor/*β*-arrestin/Erk module complex. Four out of the five individual partners of the complex (receptor, c-Src, Raf1 and MEK1) have each been docked individually on both *β*-arrestins. The corresponding complex was then constructed using these individual dockings.

## Results

### PRIOR docking algorithm

The PRIOR algorithm consists of three successive steps. First, the candidate conformations are generated using Hex^9^. Second, the top 500 solutions of Hex are reranked using a consensus scoring function. Third, different post-treatments are applied to the ranking, depending on: the functional category to which the complex belongs (either Enzyme-Substrate or Others), the *a priori* difficulty of the docking (see Material and Methods) and the pre-determined size of the solution set (5, 10, 15 or 20 conformations).

When applied to a benchmark of 108 complexes of known three dimensional structures (3D structures), Hex generated near-native solutions (see supplementary material) for 92 complexes. After rescoring and filtering, a near-native conformation is found within the 20 best-ranked solution for 91.4% of them (85 out of 92, Supplementary Table 1). This performance compares very favorably with other available methods (Supplementary Figure 1). It should be noted that for this evaluation, the scoring function was learnt in leave-one-out mode, meaning that the function used to dock one complex has been learnt on a learning set that does not contain that complex. Although negative controls would be desirable, as stated before, protein-protein docking methods are not able to predict if two proteins have sufficient affinity to interact *in vivo*, and consequently do give solutions for any pair of proteins.

**Figure 1:**
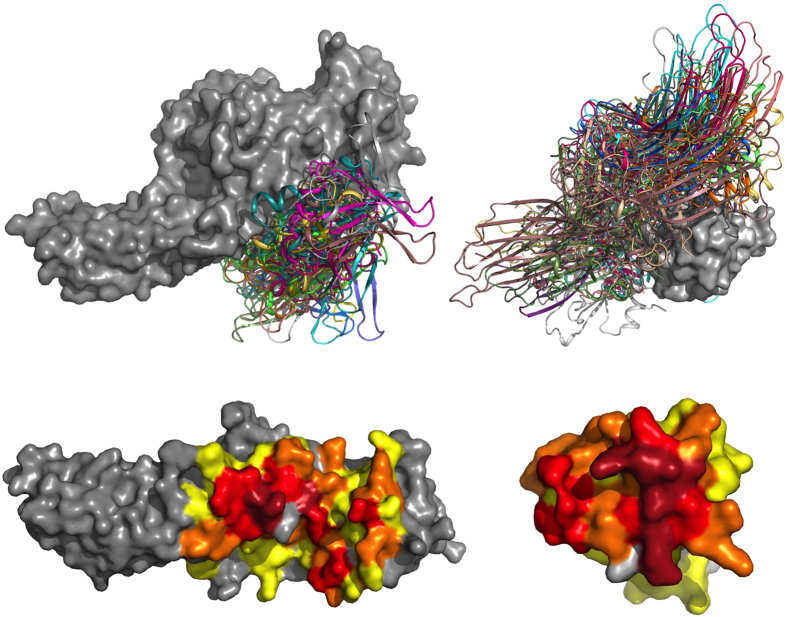
Similarity of the top 20-ranked conformations. Top: the 20 best ranked conformations of the *β*-arrestin 1/Raf1 complex have been aligned on the *β*-arrestin (left, *β*-arrestin shown in surface, Raf in ribbon) and Raf1 (right, Raf shown in surface, *β*-arrestin in ribbon) partners. Bottom: the residues of *β*-arrestin (left) and Raf (right) are colored as a function of their presence in the 20 best ranked conformations, from dark red (present in more than 15 out of 20 conformations) to yellow (present in 1 to 4 conformation), grey residues are never in the interface in these 20 conformations. These superimpositions clearly show that the interaction regions on both molecules are highly similar across these 20 conformations.

One important feature of the 20 best-ranked conformations, is that the interaction regions they represent are very similar and centered on the real regions (Fig. 1). This means that even though it is not always obvious which of the 20 is the conformation closest to the crystallographic structure, any of the 20 represents a good approximation.

Since PRIOR allows us to build near-native conformations for most heterodimers, it should be possible to build larger complexes just by assembling the docking solution heterodimers. To validate this assumption, we searched for complexes with known 3D structure, and for which the structure of the different partners isolated are also known. We found 5 such complexes (see supplementary Table 2). In each case, we chose the largest partner as the fixed protein, and docked the two other partners separately on it. We then reassembled the two dockings and compared with the crystallographic structure. In 4 cases a near-native solution was ranked within the top-20 (supplementary Table 2 and supplementary Figure 2). Contrary to previous developed methods for combinatorial docking^10^,^11^, our approach does not require the knowledge of all the partners in the complex, which makes it more versatile and more useful for the study of signaling complexes.

### Modeling the receptor/*β*-arrestin/Erk module complex

**Figure 2:**
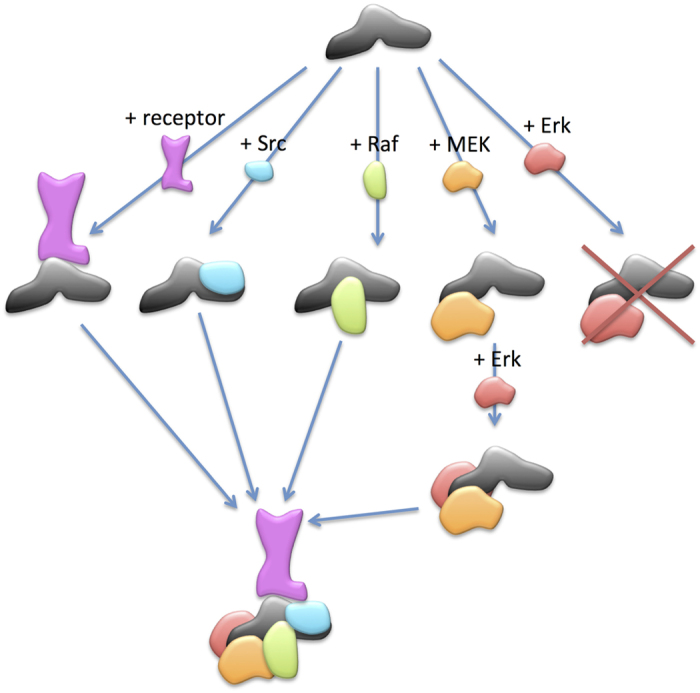
Docking models. The initial step is the docking of each partner (receptor, c-Src, c-Raf, MEK1 and ERK1) on the *β*-arrestin. During this initial step, the solutions obtained for the *β*-arrestin/ERK complex were not satisfying since they were not compatible with MEK interaction. Since ERK and MEK need to interact within the complex, resulting in ERK activation, we docked ERK on the predicted *β*-arrestin/MEK complex. The different models were then reassembled, and it should be noted that no other docking was necessary at this step since all the heterodimer models were compatible with each other.

In order to challenge the potential of our method to predict where the plethora of *β*-arrestin partners interact on the protein, we modeled a complex crucial in GPCR signaling: the receptor/*β*-arrestin/ERK module complex, containing the receptor, *β*-arrestin, c-Src, Raf, MEK and ERK. To model this complex, we docked the receptor, c-Src, Raf and MEK individually on the *β*-arrestin (Fig. 2). Each partner was docked with both *β*-arrestins, and the docking which corresponds the best to experimental data was retained. Moreover, since it is known that c-Src, Raf, Mek and Erk can bind *β*-arrestin even when attached to the receptor, we excluded any conformation placing them on the *β*-arrestin’s receptor interaction region. For each pairwise interaction, the available experimental data (regions or residues involved in the interactions) are given in Table 1.

### *β*-arrestin/c-Src interaction

**Figure 3:**
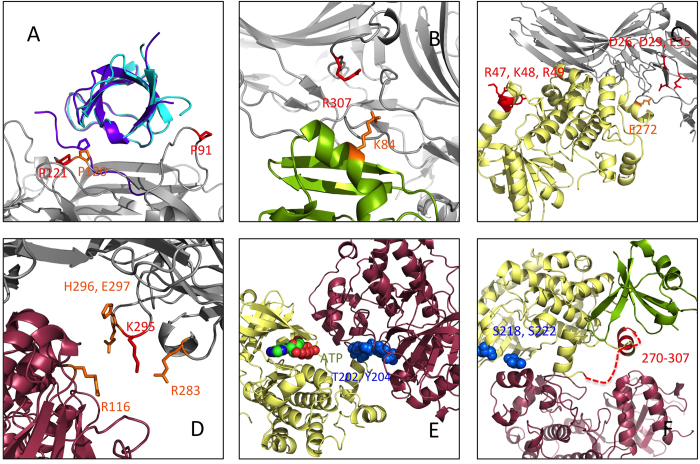
Model of the *β*-arrestin-1/ERK signaling module. Color code: *β*-arrestin in grey, c-Src in blue, Raf1 in green, Mek1 in yellow, Erk1 in dark red; experimentally identified residues are in red, other potentially important residues are in orange, phosphorylation sites are in dark blue. A: c-Src/*β*-arrestin interaction, SH3 domain and its interacting peptide of PDB 1AD5 in violet. B: Raf1/*β*-arrestin interaction. C: Mek1/*β*-arrestin interaction. D: Erk1/*β*-arrestin interaction. E: Mek1/Erk1 interaction. F: Raf1/Mek1 interaction, the region 270-307 figured by a red dashed line is unfolded.

**Figure 4:**
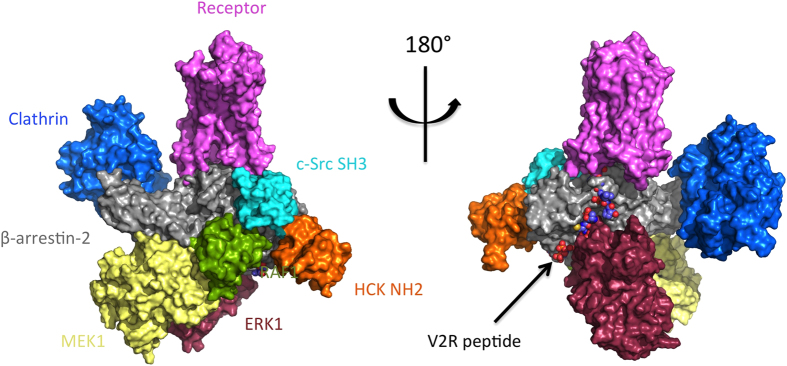
Complete complex model. The model can accommodate the presence of Clathrin and the SH2 domain of c-Src. The color code for the main complex is the same as in Fig. 3. Possible geometry of the complex including the *β*-arrestin/Erk1 module, including SH2 domain of c-Src (in orange) and clathrin (in marine blue).

We have first modeled the interaction between the two *β*-arrestins and c-Src. Since it is known that c-Src interacts with *β*-arrestin mainly through its SH3 domain^12^,^13^, only this domain was used for the docking. We have chosen to include c-Src in the complex since it has been shown that at least in some cases the *β*-arrestin-dependent ERK activation is Src-dependent^12^,^14^,^15^. Only the *β*-arrestin 2 docking gave satisfying results. In the docking model, the interaction of the SH3 domain with *β*-arrestin is very similar to the canonical interaction of SH3 domains with peptides. For example, Fig. 3A shows the superposition of a SH3 domain with its interacting peptide (PDB: 1AD5^16^). c-Src also contains SH2 and kinase domains. Although the relative orientation of the two domains is unknown, information can be deduced from the structure of Hck, a close homologue of c-Src (PDB: 3NHN^17^). Superimposing this structure onto the model of the complex shows that it can accommodate the presence of the SH2 domain without creating steric clashes (Fig. 4). Finally, there is enough free surface on *β*-arrestin in the vicinity of the SH2 domain to allow the kinase domain to contact the *β*-arrestin directly.

### *β*-arrestin/Raf1 interaction

Raf1 contains two structural domains: a kinase domain and a Ras-binding domain (RBD). To our knowledge, there is no experimental evidence showing which domain of Raf1 interacts with *β*-arrestin. Thus, we docked the two domains of Raf on the two *β*-arrestins. The kinase domain of Raf tends to dock to the receptor interaction region of both *β*-arrestins, and were thus rejected. In the docking model of the *β*-arrestin 1/Raf-RBD complex (Fig. 3B), R307 of *β*-arrestin 1, which has been shown to be an important residue for the *β*-arrestin 1/Raf interaction^18^ interacts with residue K84 in Raf 1 (Table 1). Side chain of residue K308 in *β*-arrestin 2 points in a different direction and thus does not make interactions with Raf (Supplementary Figure 3A and B).

**Figure 5:**
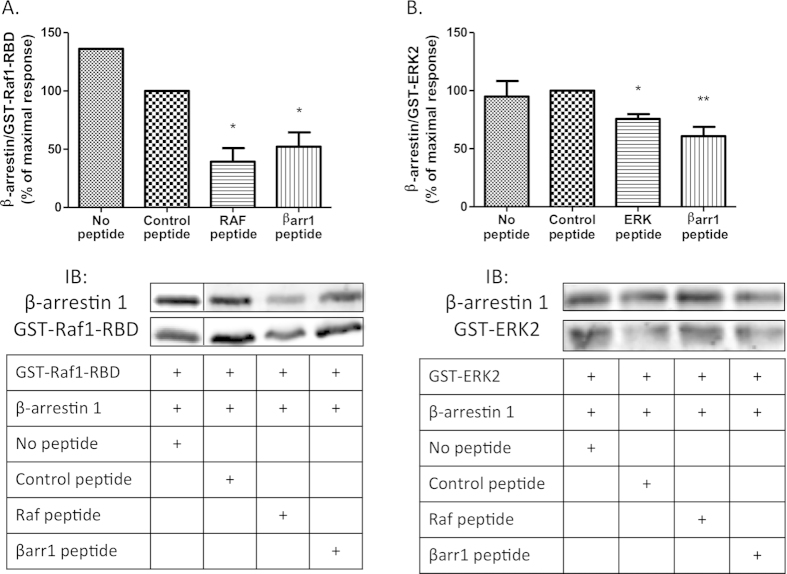
Experimental validation of the predicted *β*-arrestin-Raf1 and *β*-arrestin-ERK2 interaction sites. A) Equal amounts of GST-Raf1-RBD fusion protein were incubated with 25 ng of *β*-arrestin 1 and with or without 0.5mM of the indicated peptide. B) Representative blot for *β*-arrestin-Raf1 interaction site sequentially probed with anti-*β*-arrestin and anti-GST antibodies. B) Equal amounts of GST-ERK2 fusion protein were incubated with 25 ng of *β*-arrestin 1 and with or without 0.5mM of the indicated peptide. Representative blot for *β*-arrestin-ERK2 interaction site sequentially probed with anti-*β*-arrestin and anti-ERK antibodies. Immunoblots were quantified using Odyssey software and data were expressed as the ratio of *β*-arrestin 1 over GST-Raf1-RBD or GST-ERK respectively. The maximum response measured with the Control peptide was chosen as 100%. Data represent means ± SEM from three independent experiments. *,**: p < 0.05 specific peptides *versus* control peptides.

The interaction of the RBD domain of Raf with *β*-arrestins was demonstrated by GST-pulldown (Fig. 5). In order to validate experimentally the docking model, two peptides belonging to the interaction region were designed, one from each protein. For this design, extended regions were chosen, since a short peptide would probably fail to reproduce complex, or very constrained, secondary structures adopted in the complete structure, such as tight loops or α-helices. This explains why the peptides used were not those containing residues emphasized on Fig. 3B. Indeed, on the *β*-arrestin side, R307 lies within a tight loop, and a peptide having the same sequence would fold differently, and would thus probably not bind to Raf. Similarly, on the Raf side, K84 belongs to an α-helix, and a peptide with the same sequence will probably not fold as an helix. Using GST-pulldown experiments, we were able to demonstrate that both peptides decrease the interaction (Fig. 5), thereby confirming the prediction of both interaction regions.

### *β*-arrestin/Mek1 interaction

Mek1 is known to interact directly with *β*-arrestin^6^, and again we docked this protein with both *β*-arrestins, and the best models were obtained using *β*-arrestin 1(Fig. 3E). Whereas residue D26 might have a role in maintaining the *β*-strand in a good conformation, residues D29 and E35 make direct interactions with Mek1. Meng *et al.*^6^ have also located the interaction site on Mek1 to the 46–70 region. In our model, these residues do not make direct interactions with *β*-arrestin (see Fig. 3C). However, the distances between the side-chains and the beta strands of the C-terminal domain of *β*-arrestin is not very large (around 15 Å). Moreover, the N-terminal part of Mek is unstable and is disordered in most 3D structures. Residues S218 and S222 in Mek1 are solvent-accessible, and their location is compatible with phosphorylation by the kinase-domain of Raf1^19^ (Fig. 3G).

### *β*-arrestin/Erk interaction

The last member of the signaling complex is Erk. Erk1 showed a clear preference for the Mek binding site in both *β*-arrestins. We therefore decided to dock Erk1 on the complex *β*-arrestin 1/Mek1. The model is compatible with experimental data (Table 1, Fig. 3D), and the T202 and Y204 residues are in the vicinity of the active site of Mek1 (Fig. 3E), thus allowing phosphorylation. Xu *et al.*^20^ have shown that the *β*-arrestin 2 K285A/R286A mutant leads to lower Erk phosphorylation. In the structure of the active *β*-arrestin, these two residues form strong interactions with other *β*-arrestin residues, that are probably involved in the stabilization of the loop containing residue K294 (R295 in *β*-arrestin 1), essential for Erk interaction. Thus, our hypothesis is that these two residues do not interact directly with Erk, but are necessary for the conformation of the interaction region.

As for the *β*-arrestin/Raf docking complex validation, we designed two peptides, one from *β*-arrestin and one from Erk2, in order to experimentally validate our prediction. The GST-pulldown experiments confirmed this prediction (Fig. 5C).

### *β*-arrestin/receptor interaction

Very recently, the first structural model of the a GPCR/*β*-arrestin was published by Shukla *et al.*^21^, based on electron microscopy images, and the detection of interactions between the two proteins by mass-spectrometry. Three different docking experiments of the active *β*-arrestin on the activated *β*2-adrenergic receptor (PDB 3P0G^22^) have been made: (i) without any constraint, (ii) focusing on the receptor second intracellular loop (IL2) and the 63–75 loop of the *β*-arrestin, since these two regions have both been shown to play crucial roles in the complexation^23^,^24^,^25^,^26^,^27^,^28^, and (iii) focusing on the D78 in *β*-arrestin and K265 in the receptor, which have been shown to interact^11^. In the three experiments, very similar conformations are found at ranks 9, 4 and 7 respectively, and these are very close to the model published by Shuka *et al.* (Supplementary Figure 4). Although experiments (i) and (ii) were made before the publication by Shuka *et al.*, the results can be considered with a much higher degree of confidence with this partial validation.

### Five partner complex

As explained previously (Fig. 2) we then reassembled the different pairwise docking models. The obtained conformation shows no major steric clash, and the positions are all compatible with the insertion of the complex in the membrane (Fig. 4). Indeed, none of the modeled *β*-arrestin partner position lies in a region supposed to be occupied by the membrane. As expected, the Raf, Mek and Erk interaction regions on *β*-arrestin overlap N and C-domains (Supplementary Figure 5). The proline-rich 270-307 region of Mek1 is predicted to interact with Raf1, in agreement with experimental results of Catling *et al.*^29^ (Fig. 3F). The active site of Erk1 is in a favorable orientation for either phosphorylation of *β*-arrestin 1 residue S412^6^, or interaction with other partners (Fig. 3A,B).

## Discussion

After we had completed these docking experiments, the 3D-structure of *β*-arrestin 1 in its active conformation was published^30^. By replacing the inactive *β*-arrestin structure in our model by this new active structure, we could check that the observed conformational changes do not contradict our predictions. Moreover, the predicted epitopes are in more favorable conformations for Raf and ERK (Supplementary figure 6). Interestingly, in the active *β*-arrestin, the C-tail of the receptor takes the place of the first strand of one of the beta-sheets of *β*-arrestin N-domain. In our model, the strand was predicted to be in direct interaction with ERK. Consequently, our model predicts that in the active conformation, ERK is in direct interaction with the C-tail, introducing a receptor-dependent component to the *β*-arrestin/ERK interaction. A close-up of this interaction (Supplementary Figure 7) shows that the phosphorylation of T359 and S362 residues in the receptor might prevent the binding of ERK. Although it is not possible to align the sequences of the C-tails it seems reasonable to hypothesize that in vasopressin type 2 receptor (V2R) as in *β*2-adrenergic receptor the GRK5/6 phosphorylation sites are more proximal than the GRK2/3 ones^31^. Following this hypothesis, our model would explain why the GRK2/3 phosphorylated receptors are not capable of triggering *β*-arrestin-dependent ERK signaling, since ERK could not be recruited any more, consistent with Heitzler *et al.*, 2012^32^. As hypothesized by different authors, it can be envisioned that if the C-terminal of *β*-arrestin completely unfolds, it could occupy the channel created between Erk and Mek, and thus expose residues T383 and S361 which are known to be major regulatory phosphorylation sites 33.

As compared to the published one, our model of the receptor/*β*-arrestin/Erk complex completely accommodates the V2R peptide (Supplementary Figure 8). In particular, the most proximal phosphorylated residues in the V2R peptide (347 and 350), which can be hypothesized as being the GRK5/6 phosphorylation sites, are in interaction with *β*-arrestin whereas in Shukla *et al.* model they detach to allow the full interaction with the receptor. A tempting hypothesis is that these residues, when phosphorylated, stay in interaction with *β*-arrestin, leading to the model presented in this paper. But when unphosphorylated, as the affinity is lower, they do not bind anymore, leading to the Shukla *et al.* model. Even though highly speculative, this hypothesis would explain how two close conformations of the complex could be obtained, close enough to give similar images in electron microscopy, but different enough to explain the induced differences in output: the GRK2/3 phosphorylated complex entering in desensitization, the GRK5/6 phosphorylated one entering signaling cascades^3^,^32^.

As shown in Table 1, the only experimental data that cannot be explained with our model is the Mek1 region interacting with *β*-arrestin. However, in the model these Mek1 residues are situated at approximately 15 Å from *β*-arrestin. Using methods that allow the fine tuning of side chain orientations such as ROSETTA^34^, it should be possible to bring Mek1 closer to the *β*-arrestin and satisfy this experimental result. All the remaining experimental data confirm our model. We have also included in the complex the SH2 domain of c-Src, which position has been modeled based on the structure of the close homolog Hck (see above), and clathrin as it was described in the crystallographic structure of the complex (PDB: 3GD1^35^) (Fig. 4). From this model we conclude that the predicted interaction regions with c-Src, Raf, Mek and ERK do not overlap with the clathrin binding site, and consequently the model is compatible with internalization.

**Figure 6:**
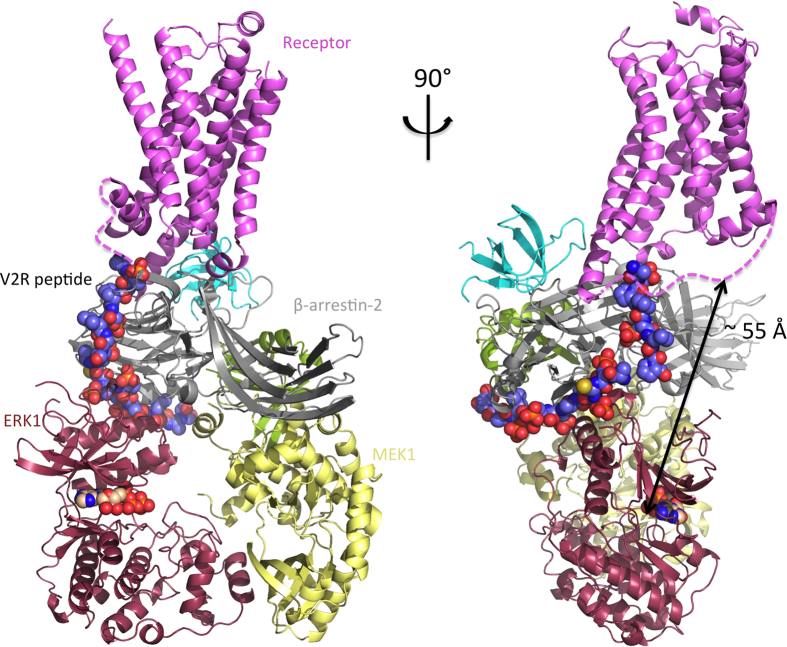
Model of the receptor/*β*-arrestin-1/ERK signaling complex, focusing on the Erk active site. Color code: *β*-arrestin in grey, c-Src in blue, Raf1 in green, Mek1 in yellow, Erk1 in dark red (including ATP shown as spheres), receptor in pink, V2R peptide in spheres.

One of the most interesting hypotheses arising from this model is that in the complex, the protein substrates of ERK would have to be close, and probably in contact with the receptor. Indeed, the active site of Erk is oriented towards the receptor, and the distance is approximately 55 Å which corresponds to the average size of a globular protein (Fig. 6). Finally, the V2R peptide is very short, only 29 residues long, but many GPCRs have much longer C-tails (72 residues for *β*2-adrenergic, 110 for α2-adrenergic, 100 for D1A dopamine receptor, etc.), and consequently the region immediately beneath the receptor could be much more crowded. Taken together these facts could explain how the substrates of ERK are receptor-dependent^36^, even though the *β*-arrestin-dependent activation pathway of ERK is common to many receptors.

## Conclusion

We have developed a new protein-protein docking method based on a coarse-grained model of protein structure and shown that taking into consideration structural and functional aspects of the individual partners and the desired size of the result set is essential. PRIOR gives near-native solutions in 80% of cases. Protein-protein complexes can be classified from their functional roles. The most homogeneous functional category contains enzyme-substrate complexes, and this is where the best results are obtained. This indicates that in the future, as the number of complexes of known structure will increase, we will be able to define more homogeneous functional classes leading to an improvement of prediction accuracy. We have also shown that this very high accuracy allows us to predict the 3D structure of multi partner complexes.

Using this method we modeled the 3D structure of the receptor/*β*-arrestin/Erk module complex. The model obtained is compatible with almost all of the available experimental data. To obtain this model we made five different dockings: *β*-arrestin + c-Src, *β*-arrestin + Raf1, *β*-arrestin + MEK, *β*-arrestin-MEK  + ERK, and *β*-arrestin + receptor and we reassembled the retained conformations. This shows that, within a large macro-molecular complex, one pairwise interaction can be accurately predicted without the knowledge of the other partners. Using this model, we designed peptides mimicking the interaction regions of the *β*-arrestin/Raf complex on each partner, and the interaction regions of the *β*-arrestin/Erk complex on each partner. In both cases we were able to disrupt the interaction experimentally, thereby confirming these predicted interaction regions. The model is also compatible with the interaction with clathrin, and consequently internalization. Finally, the model leads to interesting hypotheses which could explain why GRK2/3 phosphorylated receptors do not produce *β*-arrestin dependent phosphorylated ERK, or how receptor specificity is translated into specificity of *β*-arrestin-activated ERK for selective substrates.

Overall, these results show that our analysis pipeline is especially pertinent for understanding the interactome of hub proteins, which play crucial roles in cells. This also shows that despite a very large number of partners, the hub protein binds to each of them with great specificity, and the interaction regions can be efficiently predicted from the structures of isolated partners. In particular this should help to determine which proteins are in competition for the same site and mutually exclusive (XOR), which importance was demonstrated recently^37^, and could explain the differences in relative abundance of proteins between different cell types. Finally, such analysis pipeline will also help the analysis of high-throughput data such as phosphoproteomics or interactomics experiments by helping to identify the direct and indirect interactions, and helping to design high value experiments.

## Methods

### Generation of candidate conformations

The candidate conformations were generated using Hex^9^, with clustering threshold set to 9.0 Å Root Mean Squared Deviation (RMSD), and relative orientations of the two partners restricted to 4523 from the most favorable orientation found by Hex.

We distinguished 4 categories of near-native conformations. Three of these categories were used as defined in the CAPRI experiment^38^ (High quality, medium quality, Acceptable), to which we added a fourth category, Correct:

- High quality (4): fnat > 0.5 AND (Irmsd < 1 OR Lrmsd < 1);
- Medium quality (3): [fnat 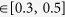 AND (Irmsd < 2 OR Lrmsd < 5)] OR [fnat > 0.5 AND Irmsd > 1 AND rmsd > 1];
- Acceptable (2): [fnat > 0.3 AND Irmsd > 2 AND Lrmsd > 5] OR [fnat 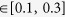 AND (Irmsd < 4 OR rmsd < 10)];
- Correct (1): fnat < 0.3 AND (Irmsd < 5 OR Lrmsd < 10);

Here fnat is the fraction of native contacts that is predicted, Irmsd is the root mean square deviation between the model and the native structure restricted to the interface amino acids, and Lrmsd for the complete Ligand chain, which is by convention the smallest partner, the Receptor chains of the model and the native structure being superimposed. Conformations not belonging to any of these 4 categories are named incorrect. Conformations belonging to one of these categories are named near-native.

### Training attributes and learning sets

The Voronoi-based coarse-grained model and attributes are defined in^39^. Each attribute was normalized using:
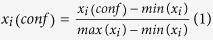
where *min*(*x*_*i*_) and *max*(*x*_*i*_) represent respectively the minimum and maximum values observed for attribute *x*_*i*_ in the learning set. Missing values were replaced by the maximal value observed in the learning set for the same attribute, except for the functions trained on Enzymes for which a missing value was replaced by the median of all values observed on the set for the same attribute.

The positive learning set originates from the one defined in^39^, enriched with benchmark 4.0 complexes not already present, leading to 249 bound-unbound or unbound-unbound complexes with known three-dimensional structures. From Hex results, and for each complex, the 10 best-ranked non-native examples were retained as negative examples. The positive and negative sets were divided in 4 different sets (2 positive ones and 2 negative ones) depending on the functions as defined in the Benchmark: Enzyme (E) for enzyme-substrate and enzyme-inhibitor (82 complexes), and Others (167 complexes, includes Antibodies). Scoring functions were learnt either on the complete sets (non-specific function), or on specific sets. The consensus score between specific and non-specific functions, based on the random-energy model, was computed as follows:
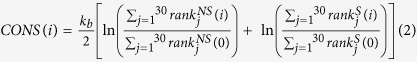
where 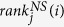 is the rank of conformation *i* under the non-specific function number *j* (30 functions), 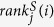 is the rank of conformation *i* under the specific function number *j* (30 functions). The ranks of the best-ranked conformation were used for normalization.

For each complex of the benchmark, the scoring functions were learnt using a leave-one-out procedure (the complex itself and all the negative examples derived from it were excluded from the learning sets).

Our previous studies have led us to reconsider the level of difficulty indicated in the benchmark, which did not correlate well with our ability to correctly predict the structure of the complex. Moreover, this difficulty level relies on the differences between bound and unbound conformations, and is thus not accessible for unknown complexes. After a thorough analysis of different structural features of the individual partners, we have concluded that the most important factors that impair the prediction accuracy are: the multimeric state of individual partners; the presence of a solvent-accessible cofactor; and the flexibility of the partners as evaluated by comparing the existing structures of the same protein or close homologs. We therefore designed a new classification according to these factors. A complex presenting none of these factors was classified as easy (36 complexes), a complex presenting one of them was classified as medium (44 complexes), and a complex presenting two or more was classified as difficult (12 complexes).

### Scoring functions and post-treatments

Using training attributes described in^39^, genetic algorithms were used to parameterize a set of functions to discriminate true positive from false positive examples. The scoring functions learnt in the present study have the form:
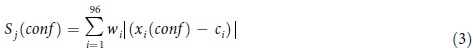
where for each learning attribute *x*_*i*_, *w*_*i*_ and *c*_*i*_ are the weight and centering value respectively, optimized by learning procedures.

The fitness function used in the genetic algorithms is the area under the ROC curve (Receiver-Operator-Characteristic). A λ + *μ* scheme has been used, with λ *=* 20 parents *μ* *=* *120* children, the maximal number of generations was set to 500, and classical cross-over operator and auto-adaptative mutation were used. Following the genetic algorithm optimization, the resulting parameter sets (weights and centering values) were further optimized using CMA-ES (Covariance Matrix Adaptation - Evolution Strategy)^40^, using the same fitness function, and a maximal number of 1,000 iterations. The functions were learnt on the full dataset and on each of the specific datasets (Enzymes and Others). Finally, the consensus score between specific and non-specific functions was computed using the random-energy model (see supplementary material).

Given the Hex ranking for a complex, each amino acid *i* of both partners was assigned a value *V*_*i*_ computed as the sum of the ranks of the candidate conformations in which this amino acid belongs to the interface, using a given scoring function. For each candidate conformation, the A-score is the sum of the *V*_*i*_. For each complex and for each candidate conformation, the C-score was computed as the sum of its own rank and the ranks of the other candidate conformations sharing an RMSD lower than 5 Å^2^ with it, and which ranks are higher than its own rank. Different combinations of the A-score, C-score, Hex rank and consensus rank were used depending on the functional category, the a priori difficulty and the chosen size of the result set (supplementary Table 1).

After ranking and post-treatment, one last filter was applied: each conformation was compared to each conformation having lower rank, which was eliminated if it shared a RMSD lower than 5 Å^2^ with the first one.

### Method evaluation and statistics

For testing purposes, the protein-protein docking benchmark v4.0^41^ was used. All the complexes with more than two chains were removed since our method was originally designed only for dimers^42^. This resulted in a list of 108 complexes. For statistical tests, since in most cases we compared distributions including small effectives (below five), and linked series, we used the test described in^43^: given two methods *A* and *B*, applied to two sets of *n*_*A*_ and *n*_*B*_ examples, not necessarily different, the probability of observing numbers of successes *a* and *b* under the null hypothesis *P*_*A*_ = *P*_***B***_ is:
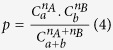


The null hypothesis can be rejected with a risk *α* if *p* *<* *α*.

### Docking of the signaling complex

Docking of the signaling complex was conducted using the method described herein. The PDB structures used were: 1ZSH^44^ for *β*-arrestin 1, 3P2D^45^ for *β*-arrestin 2, 1FMK^46^ for c-Src, 4JQI for active *β*-arrestin^30^, 1GUA^47^ for Raf1, 3EQB^48^ for Mek1, 2ZOQ^49^ for Erk1, 3SN6^50^ for adrenergic receptor. Although the association of Erk2 is usually stronger than that of Erk1, the Erk1 structure was preferred over the Erk2 structure since there are some extra-residues in both N and C-ter, moreover, these structures are very similar (0.6 Å RMSD). ATP was modeled in the active site of Erk1 by superimposition of 3EQB on 2ZOQ. Images were generated using Pymol.

### GST production and pull-down assay

Raf1-RBD cDNA inserted in pGEX was kindly donated by Dr V. Fafeur (Institut de Biologie de Lille, France) and GST-ERK2 purified protein was provided by Interchim (Montluçon, France). GST-Raf1-RBD and GST-ERK2 fusion proteins were expressed in NEB 5-alpha competent Escherichia coli (New England Biolabs, Ipswish, MA, USA), as previously described^51^. GST alone was used as a control. Purification was performed on Glutathione Sepharose 4B beads (GE Healthcare, Buckinghamshire,UK) and aliquots were stored at –20 °C. GST pull down assays were carried out using human recombinant *β*-arrestin 1 (Gentaur, San Jose, CA, USA) as the prey. Interfering peptides were designed according to the predicted docking and were synthesized by Genecust laboratories (Dudelange, Luxembourg). They corresponded to 9 and 10 amino acid-long sequences located at the interaction interface of either *β*-arrestin 1, Raf1 or ERK2. The control peptides corresponded to either a sequence of *β*-arrestin 1 located outside of the predicted interaction site or a sequence of a completely different protein (see Table 2). Equal amounts of immobilized GST fusion proteins or GST were incubated with 25 ng of human recombinant *β*-arrestin 1 and 0.5 mM of one of the peptides in a binding buffer containing 20 mM Hepes pH 7.9, 50 mM KCl, 2.5 mM MgCl2, 1 mM DTT, 10% glycerol and protease and phosphatase inhibitor cocktail (Thermo Scientific, Waltham, MA, USA) for 1 hour at 4 °C. GST beads were then washed six times in binding buffer and resuspended in 25*μ*L of 2X Laemmli buffer. Equal amounts of samples were separated in a 10% acrylamide gel by SDS-PAGE. Membranes were incubated overnight with polyclonal anti-*β*-arrestin primary antibody (1: 2,000, Abcam, Cambridge, England) and then with IRDye Alexa fluor 680 secondary antibody (1: 15,000, Licor, Lincoln, NE, USA). Protein loading homogeneity was confirmed by stripping, washing and reprobing membranes with polyclonal anti-GST antibody for GST-raf1-RBD (1: 500, Life technologies, Carlsbad, CA, USA) and with anti-ERK antibody for GST-ERK2 (1: 10 000, Santa Cruz Biotechnology, Santa Cruz, CA, USA). Imaging and quantications were carried out using an ‘Odyss’ey CLX scanner (Licor).

### Statistical analysis

Statistical analysis of the data were performed with one-way ANOVA (Bonferroni’s multiple-comparison tests) to compare samples using PRISM software.

## Additional Information

**How to cite this article**: Bourquard, T. *et al.* Unraveling the molecular architecture of a G protein-coupled receptor/β-arrestin/Erk module complex. *Sci. Rep.* **5**, 10760; doi: 10.1038/srep10760 (2015).

## Supplementary Material

Supplementary Information

## Figures and Tables

**Table 1:** Known interactions in the receptor/*β*-arrestin/ERK module complex. Interacting amino acids present in the models or compatible are shown in bold, those not found and not easily compatible are shown in italic.

**Partner 1**	**Partner 2**	**Residues/Regions**	**Ref. and remarks**
*β*-arrestin 2	receptor	**L68 V70 L71 L73 V167** *L191 S234 Y238* **T246**	23
*β*-arrestin 1	receptor	**Finger, middle and lariat loops**	21
*β*-arrestin 1	receptor	**K77 D78**	21
Receptor	*β*-arrestin 1/2	**IL2 IL3**	51,52
Receptor	*β*-arrestin 1/2	**K265**	21
*β*-arrestin 1	c-Src	**P91 P121**	12,53
c-Src	*β*-arrestin 1	**SH3 domain**	12,53
*β*-arrestin 1	Raf1	**R307**	18
*β*-arrestin 1 & 2	Raf1	**N-domain C-domain**	8
*β*-arrestin 1 & 2	MEK1	**N-domain C-domain**	8
*β*-arrestin 1	MEK1	**D26 D29**	6
MEK1	*β*-arrestin 1	*46-70 R47, K48, R49*	6
*β*-arrestin 2	ERK1	**K295**	20
*β*-arrestin 1 & 2	ERK1	**N-domain C-domain**	8
ERK1	MEK1	**T202 Y204**	phosphorylated by MEK1
MEK1	Raf1	**270-307**	29
MEK1	Raf1	**S218 S222**	19 phosphorylated by Raf1

**Table 2:** Peptides used in competition experiments.

**Name**	**Sequence**	**Resuspended in**
Control_Raf1	H-DVLGLSFRK-OH	H_2_O
Raf1	H-KQRTVVNVRN-OH	H_2_O
*β*arr1_Raf1	H-HIDLVEPVD-OH	H_2_O
Control_ERK2	H-VRGLGPSYCSFG-OH	H_2_O
ERK	H-ISPFEHQTY-OH	H_2_O + 5% DMSO
*β*arr1_ERK2	H-LKHEDTN-OH	H_2_O
