# Supplementary material for: Unraveling the molecular architecture of a G protein-coupled receptor/β-arrestin/Erk module complex
Source: Sci Rep. 2015 Jun 1;5:10760. doi: 10.1038/srep10760 (PMC4649906; doi:10.1038/srep10760)
Supplement: Supplementary Information [file srep10760-s1.pdf]

# Supplementary information - Unraveling the molecular architecture of a G-protein coupled receptor/$\beta$-arrestin/Erk module complex.

Thomas Bourquard[1,2], Flavie Landomiel[1], Eric Reiter[1], Pascale Crépieux[1], David W. Ritchie[2], Jérôme Azé[3], and Anne Poupon[1,*]

[1]BIOS group, INRA, UMR85, Unité Physiologie de la Reproduction et des Comportements, F-37380 Nouzilly, France; CNRS, UMR7247, F-37380 Nouzilly, France ; Université François Rabelais, 37041 Tours, France; IFCE, F-37380 Nouzilly, France.
[2]INRIA Nancy, 615 Rue du Jardin Botanique, 54600 Villers-lès-Nancy, France.
[3]Bioinformatics group – AMIB INRIA – Laboratoire de Recherche en Informatique, Université Paris-Sud, 91405 Orsay, France
[*]Correspondance to Anne.Poupon@tours.inra.fr

April 2, 2015

## Comparing the performances of PRIOR with that of existing methods

The performance of PRIOR was compared with that of other available methods by counting the number of test cases for which a near-native solution is ranked within the 20 best-ranked conformations. We compared these values for the cases common to a given method's test set and PRIOR's test set on the one hand, and for each method's complete test set on the other hand (Figure 1). When comparing results obtained on the same test sets or on complete sets, PRIOR gives the highest values. Percentages obtained by RosettaDock are surprisingly low. However, it should be reminded that, unlike all other methods, RosettaDock does not rank a large number of conformations, but rather optimizes just one given conformation.

Overall, PRIOR brings a very clear improvement of the ability to predict the conformations of protein-protein complexes.

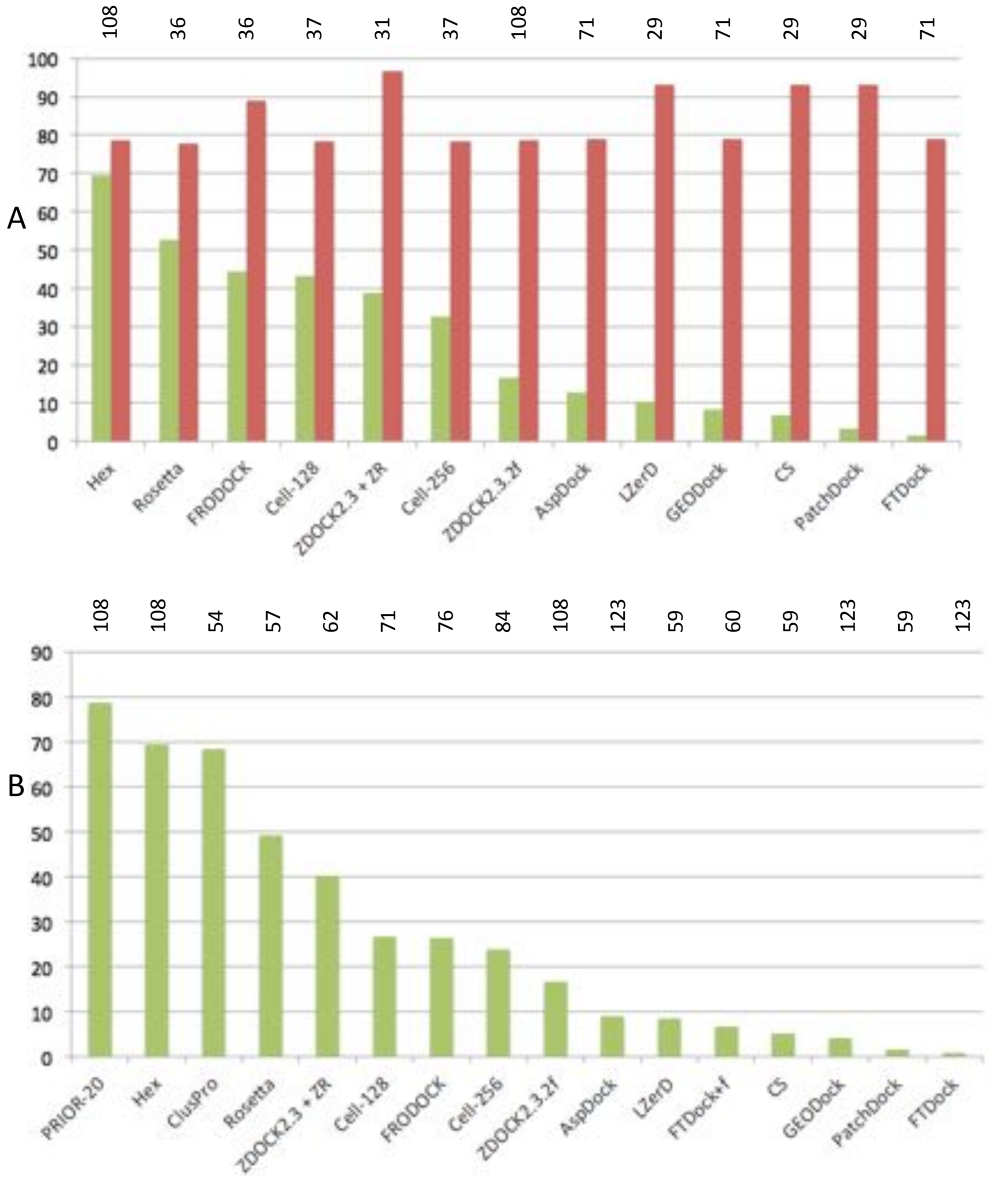


Figure 1: Percentages of complexes for which a near-native conformation is found within the 20 best-ranked conformations for different protein-protein docking methods. A: for each method, comparison of the percentages obtained by the considered method and PRIOR on test cases common to both test sets. Number of such test cases are indicated above the histogram for each method, only methods for which the number of such cases is at least 20 were considered. B: percentages on complete test sets. Only methods tested on at least 50 complexes were considered. Number of examples in each test set is indicated above the histogram. Tested methods: Hex [1], Rosetta [2], FRODOCK [3], Cell-dock (Cell-128 and Cell-256) [4], ZDOCK2.3 + ZR (ZDock2.3 with ZRank) [5], ZDOCK2.3.2f (ZDOCK2.3.2 with advanced 3D convolution library) [6], FTDock+f (FTDock with residue conservation information) [7], AspDock [8], LZerD [9], GEODock [8], CS (Context shapes) [9], PatchDock [9] and FTDock [7].

Table 1: Number of complexes with near-native solution ranked in top N (N=5, 10, 15 and 20) for different categories of complexes and different levels of difficulty (D: Difficult, M: Medium, E: Easy). H: Hex, CONS: consensus, A: A-score, C: C-score, SA: consensus + A-score, SC: consensus + C-score, SH: CMA-CONS + Hex rank, SAC: consensus + A-score + C-score, SAH: consensus + A-score + Hex rank, SCH: consensus + C-score + Hex rank, SACH : consensus + A-score + C-score + Hex rank, minSH : minimum of consensus and Hex rank. test Hex and test CONS: statistical test of using the different methods for the different categories against using either Hex or consensus for all, ⋆: significative at 10%, ⋆⋆: significative at 5%. Computed on the 92 complexes of the benchmark v4.0 for which Hex generated near-native solutions.

Enzymes (40)

| | D (3) | | M (12) | | E (25) | | Total | |
|---|---|---|---|---|---|---|---|---|
| top 5 | minSH | 1 | SC | 10 | SA | 20 | 31 | 77.5 |
| top 10 | minSH | 2 | minSH | 11 | SAH | 23 | 36 | 90 |
| top 15 | minSH | 2 | H | 12 | SAH | 24 | 38 | 95 |
| top 20 | minSH | 2 | H | 12 | A | 25 | 39 | 97.5 |

Others (52)

| | D (9) | | M (32) | | E (11) | | Total | |
|---|---|---|---|---|---|---|---|---|
| top5 | CONS | 6 | SC | 22 | minSH | 8 | 36 | 69.2 |
| top 10 | minSH | 7 | SC | 25 | minSH | 9 | 41 | 78.8 |
| top 15 | minSH | 7 | SACH | 27 | minSH | 10 | 44 | 84.6 |
| top 20 | SF | 8 | SACH | 28 | minSH | 10 | 46 | 88.5 |

Total (92)

| | D (12) | M (44) | E (36) | Total | % | | test Hex | test CONS |
|---|---|---|---|---|---|---|---|---|
| top 5 | 7 | 32 | 28 | 67 | 72 | | ⋆⋆ | ⋆ |
| top 10 | 9 | 36 | 32 | 77 | 82.8 | | ⋆⋆ | ⋆ |
| top 15 | 9 | 39 | 34 | 82 | 88.2 | | ⋆⋆ | ⋆ |
| top 20 | 10 | 40 | 35 | 85 | 91.4 | | ⋆⋆ | ⋆⋆ |

## Multi-partner complexes

Table 2: Multi-partner complexes. Near-native conformations ranked within the top-20.

| Complex | PDB | Capri | Rank | Lrmsd | Irmsd | fnat |
|---|---|---|---|---|---|---|
| Actin / Deoxyribonuclease-1 / Gelsolin | 3CJC | Medium | 1 | 9.12 | 4.56 | 0.5 |
| | | Medium | 2 | 3.66 | 1.95 | 0.89 |
| | | Medium | 3 | 5.7 | 3.27 | 0.55 |
| | | Medium | 7 | 3.51 | 1.98 | 0.66 |
| Von Willebrand factor / Botrocetin / Platelet glycoprotein Ib | 1U0N | Incorrect | | | | |
| Transport protein particle (TRAPP) I subunits 1, 3, 4, 6A | 2J3T | Acceptable | 13 | 15.37 | 5.49 | 0.31 |
| Urokinase-type plasminogen activator / Urokinase plasminogen activator surface receptor / ATN-615 anti-uPAR antibody | 2FD6 | Acceptable | 4 | 23 | 20 | 0.32 |
| | | Acceptable | 10 | 20.45 | 23.52 | 0.42 |
| | | Acceptable | 13 | 8.47 | 8.01 | 0.11 |
| | | Acceptable | 16 | 24.75 | 25.95 | 0.42 |
| Urokinase-type plasminogen activator / Urokinase plasminogen activator surface receptor / Vibronectin | 3BT1 | Medium | 13 | 3.1 | 3.6 | 0.37 |

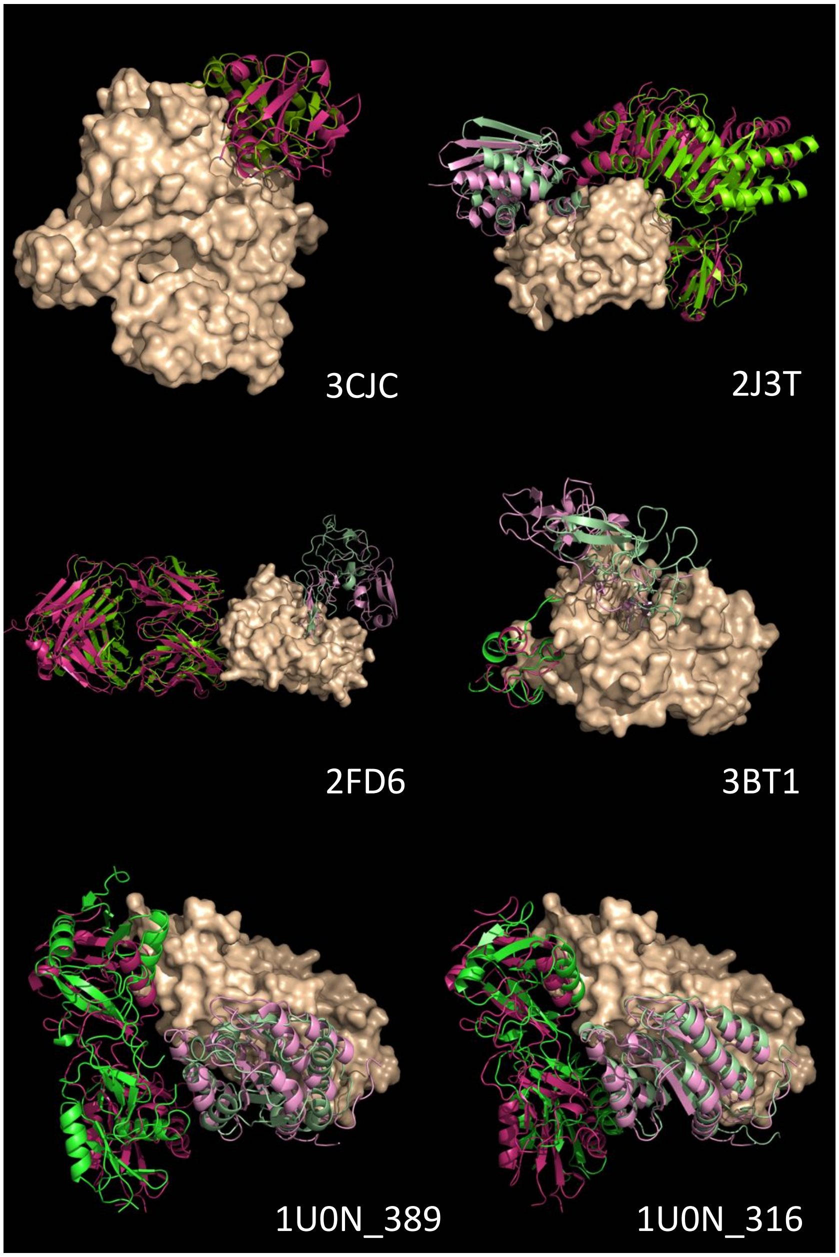


Figure 2: Docking solutions obtained for the five multimers deriving from the benchmark. For 1U0N the solutions obtained starting from docking models of the complex 1M10 number 389 (ranked 2, RMSD 9.53) and 316 (ranked 31, RMSD 2.04) are presented. The receptor, which is by convention the largest of the two initial chains is shown in surface. The ligand of the initial docking is shown in light pink (3D structure) and light green (model), and the ligand of the secondary docking is shown in pink (3D structure) and green (model).

## Docking models

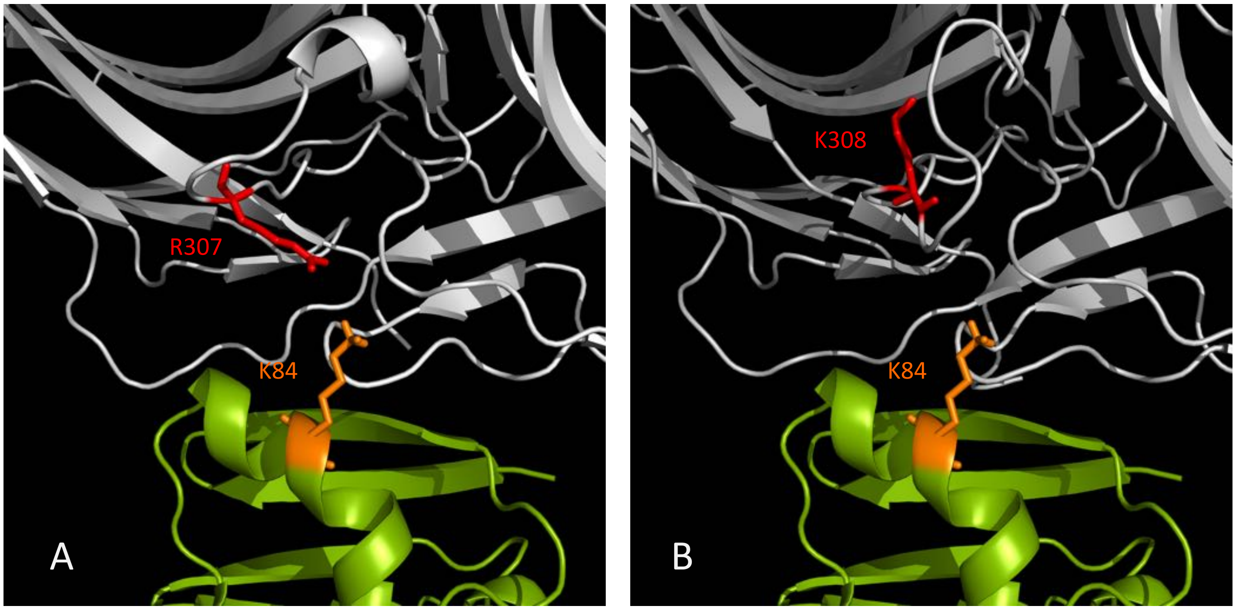


Figure 3: In $\beta$-arrestin 1, residue R307 was shown to be crucial for the interaction with Raf, whereas equivalent residue K308 in $\beta$-arrestin 2 does not affect binding. In our models, residue R307 does make an interaction with residue K84 in Raf. In $\beta$-arrestin 2, side chain of residue K308 points upward and thus does not make any interaction with Raf.

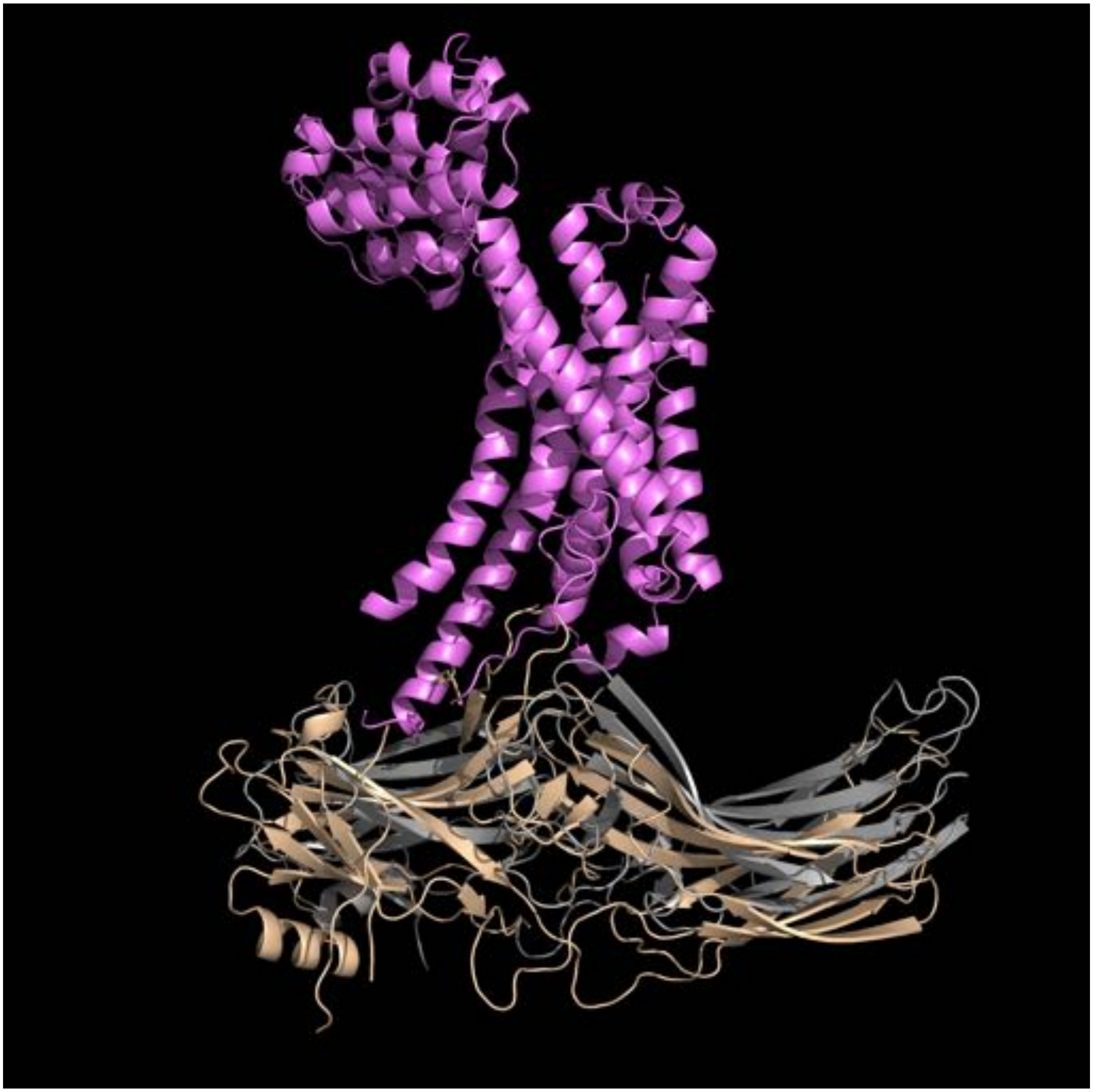

Figure 4: Superposition of the $\beta$-arrestin/receptor complex models obtained in this work (grey) and published by Shukla *et al.* (light brown [10], receptor in pink).

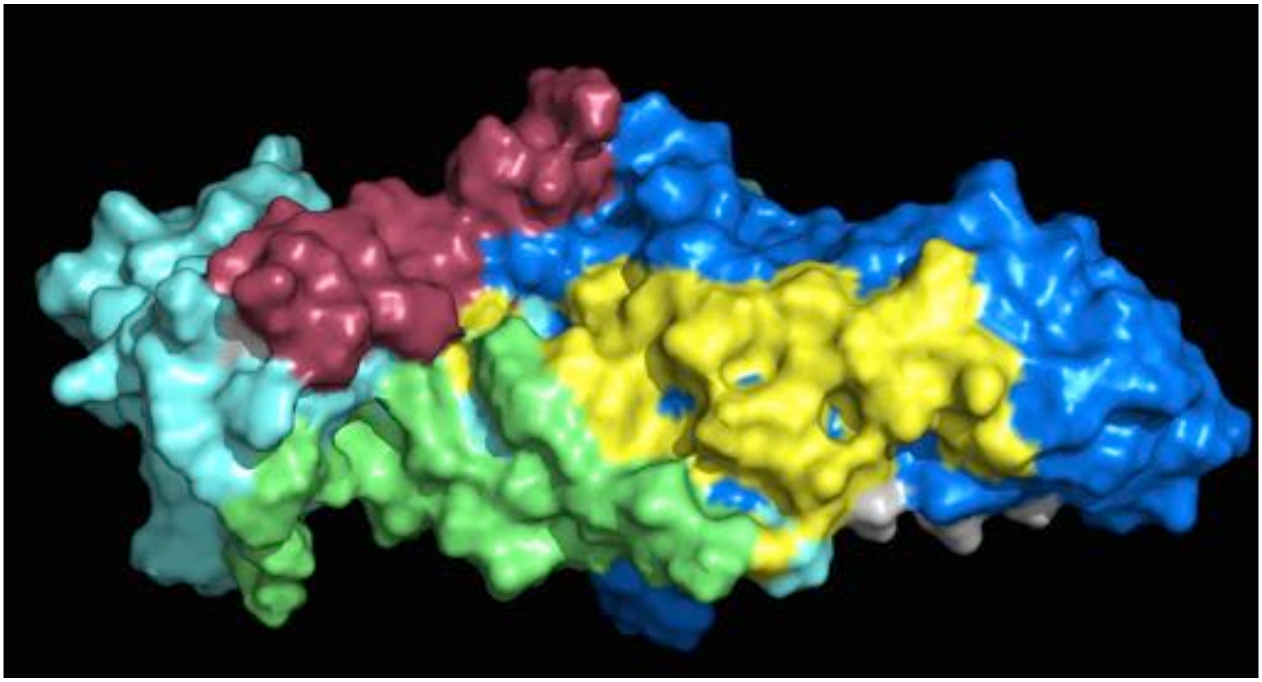

Figure 5: Interaction regions on $\beta$-arrestin for Raf (green), Mek (yellow) and Erk (Purple) overlap the N-half (light cyan) and C-half (blue).

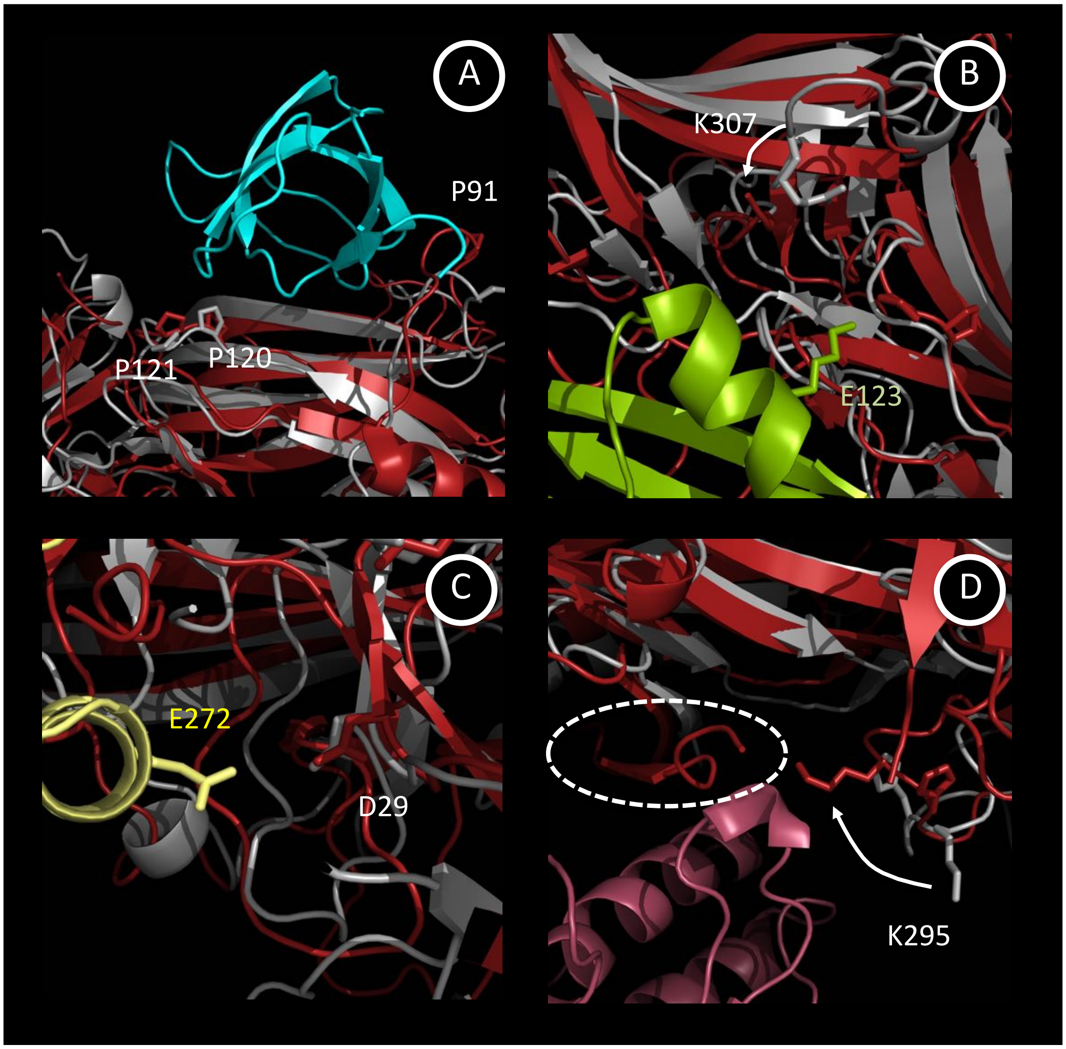


Figure 6: Details of the predicted interactions with active $\beta$-arrestin. Inactive $\beta$-arrestin in grey, active $\beta$-arrestin in red. A: the positions of P120 and P121 belonging to the c-Src epitope on $\beta$-arrestin are slightly affected by the activation, residue P91 is much closer to c-Src in the active conformation. B: residue K307, which is known to be important for the interaction with Raf is not present in the active $\beta$-arrestin structure. However, from the position of residue 308, it can be deduced that K307 will be closer to the predicted position of Raf in the active conformation than in the inactive one. C: The conformation of the Mek epitope on $\beta$-arrestin is slightly affected by the activation. D: the loop containing residue K295 undergoes important conformational changes upon activation, which brings it much closer to the predicted position of ERK1, allowing direct interactions. It should also be noted that the C-tail of the receptor is predicted to come in direct interaction with ERK1.

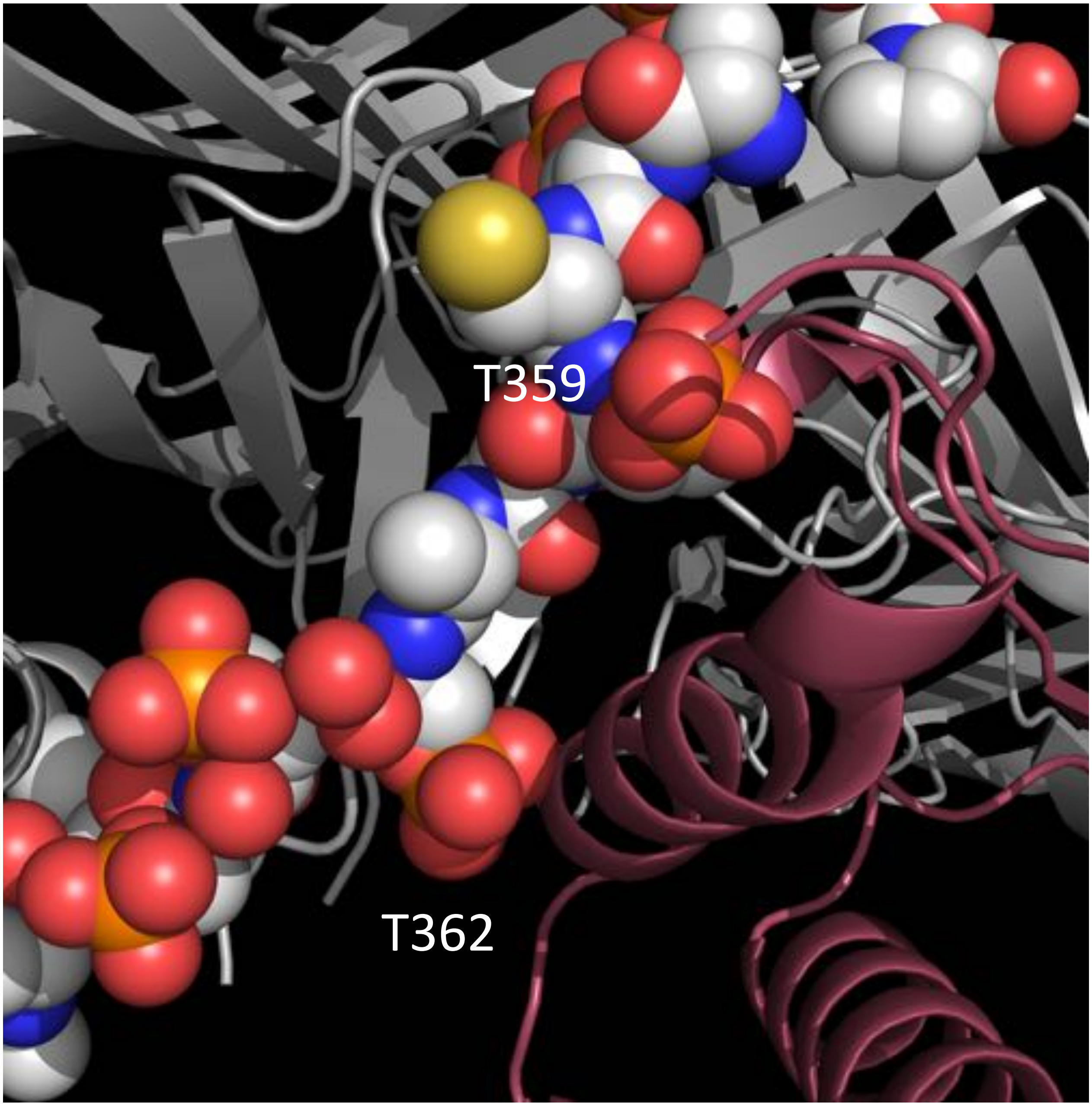


Figure 7: Detail of the interactions between the active $\beta$-arrestin, the V2R peptide and ERK. $\beta$-arrestin is shown in grey, ERK in dark red, and the V2R peptide is shown as spheres.

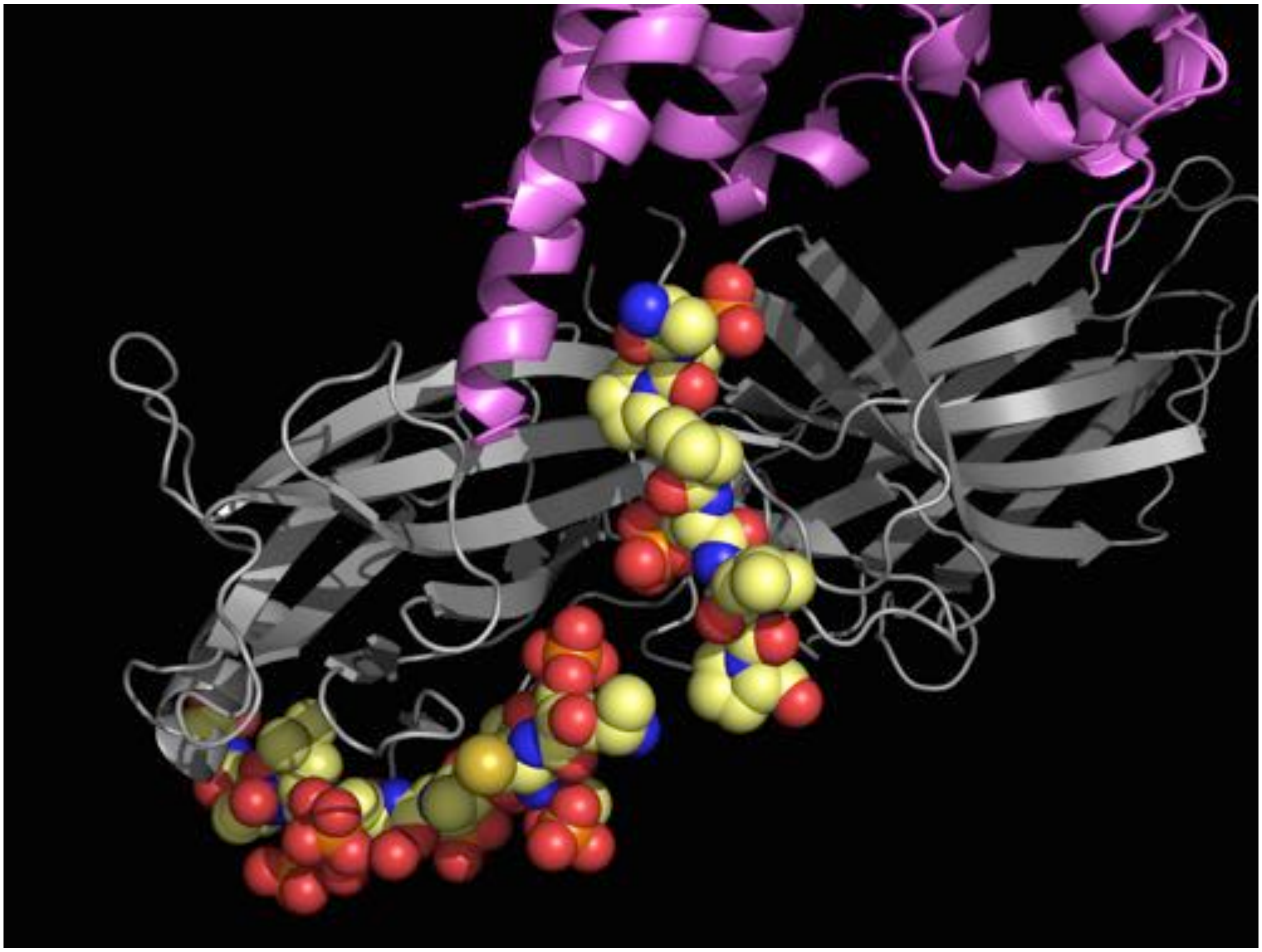

Figure 8: Position of the V2R peptide (spheres) in the docking model of the $\beta$-arrestin (grey)/receptor (pink) complex.

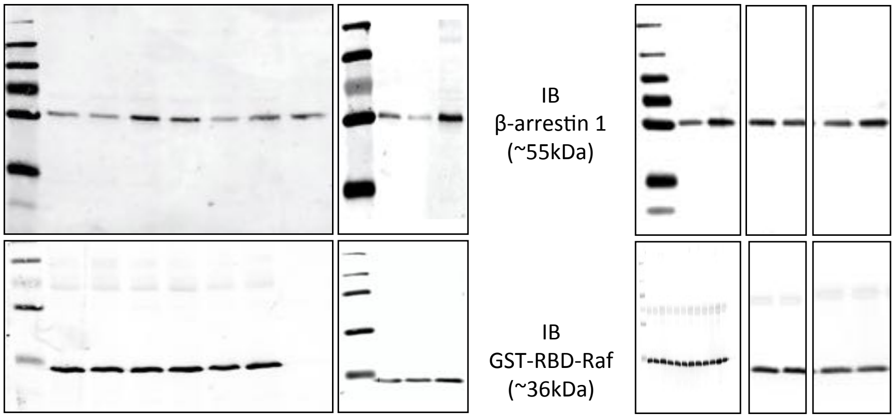


| *Replicate #* | *1* | | | *2* | | | */* | *3* | | |
|---|---|---|---|---|---|---|---|---|---|---|
| GST-Raf1-RBD | + | + | + | + | + | + | | + | + | + |
| βarr1 | + | + | + | + | + | + | + | + | + | + |
| Control peptide | + | | | + | | | | + | | |
| Raf peptide | | + | | | + | | | | + | |
| βarr1 peptide | | | + | | | + | | | | + |

| *Replicate #* | *1* | | *2* | | *3* | |
|---|---|---|---|---|---|---|
| GST-Raf1-RBD | + | + | + | + | + | + |
| βarr1 | + | + | + | + | + | + |
| Control peptide | | + | | + | | + |

Figure 9: Experimental validation of the predicted $\beta$-arrestin1-Raf 1 interaction sites. Equal amounts of GST-Raf1-RBD fusion protein were incubated with 25 ng of $\beta$-arrestin1 and with or without 0.5 mM of the indicated peptide. On the left side, three independent experiments comparing the effects of control, Raf and barr1 peptides were blotted and sequentially probed with anti-$\beta$-arrestin (upper blots) and anti-GST (lower blots) antibodies. On the right side are shown three independent experiments comparing the effect of control peptide versus no peptide on the direct interaction of $\beta$-arrestin1 with GST-Raf1-RBD. All these blots were quantified and normalized as presented in figure 5A.

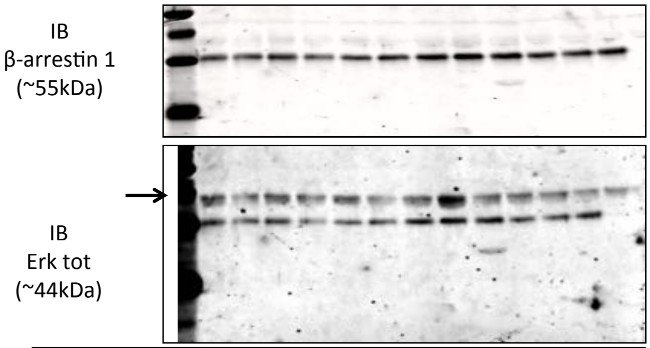


| *Replicate #* | *1* | | | | *2* | | | | *3* | | | | */* |
|---|---|---|---|---|---|---|---|---|---|---|---|---|---|
| GST-ERK2 | + | + | + | + | + | + | + | + | + | + | + | + | |
| βarr1 | + | + | + | + | + | + | + | + | + | + | + | + | + |
| Control peptide | | + | | | | + | | | | + | | | |
| ERK peptide | | | + | | | | + | | | | + | | |
| βarr1 peptide | | | | + | | | | + | | | | + | |

Figure 10: Experimental validation of the predicted $\beta$-arrestin1-ERK2 interaction sites. Equal amounts of GST-ERK2 fusion protein were incubated with 25 ng of $\beta$-arrestin1 and with or without 0.5 mM of the indicated peptide. Three independent experiments were simultaneously blotted and sequentially probed with anti-$\beta$-arrestin (upper blot) and anti-ERK (lower blot) antibodies. The arrow shows the $\beta$-arrestin signal that remained after the blot was stripped and reprobed with anti-ERK antibody. These blots were quantified and normalized as presented in figure 5B.

# References


[1] Ritchie, D. and Venkatraman, V. *Bioinformatics* **26**(19), 2398–405 (2010).

[2] Chaudhury, S., et al. *PLoS One* **6**(8), e22477 (2011).

[3] Garzon, J. I., et al. *Bioinformatics* **25**(19), 2544–2551 Oct (2009).

[4] Pons, C., et al. *Bioinformatics* **28**(18), 2394–2396 Sep (2012).

[5] Pierce, B. and Weng, Z. *Proteins* **67**(4), 1078–1086 Jun (2007).

[6] Pierce, B. G., Hourai, Y., and Weng, Z. *PLoS One* **6**(9), e24657 (2011).

[7] Duan, Y., Reddy, B. V. B., and Kaznessis, Y. N. *Protein Sci* **14**(2), 316–328 Feb (2005).

[8] Li, L., Guo, D., Huang, Y., Liu, S., and Xiao, Y. *BMC Bioinformatics* **12**, 36 (2011).

[9] Venkatraman, V., Yang, Y. D., Sael, L., and Kihara, D. *BMC Bioinformatics* **10**, 407 (2009).

[10] Shukla, A. K., et al. *Nature* Jun (2014).

[11] Mendez, R., Leplae, R., Lensink, M. F., and Wodak, S. J. *Proteins* **60**(2), 150–69 (2005).
